# Pencil beam excitation using a 2D spatially selective adiabatic T2-prep for imaging the left coronary arterial system

**DOI:** 10.1186/1532-429X-16-S1-W34

**Published:** 2014-01-16

**Authors:** Andrew J Coristine, Matthias Stuber

**Affiliations:** 1 Department of Radiology, Vaudois University Hospital Centre (CHUV)/University of Lausanne (UNIL), Lausanne, Switzerland; 2 CardioVascular Magnetic Resonance (CVMR) research centre, Centre for BioMedical Imaging (CIBM), Lausanne, Switzerland

## Background

2D spatially selective radiofrequency (RF) pulses can be used to constrain the location from which an MR signal is obtained. This may lead to more time-efficient data collection by reducing the field of view (FoV) or may improve image quality by suppressing artefacts from outside the area of interest. Meanwhile, T2-Preparation, or T2-Prep, is a magnetization preparation scheme used to improve blood/myocardium contrast. We propose incorporating a "pencil-beam" 2D RF pulse into a T2-Prep module, so as to produce a "2D T2-Prep" that combines T2-weighting with an intrinsic spatial selectivity.

## Methods

**Figure 1:**
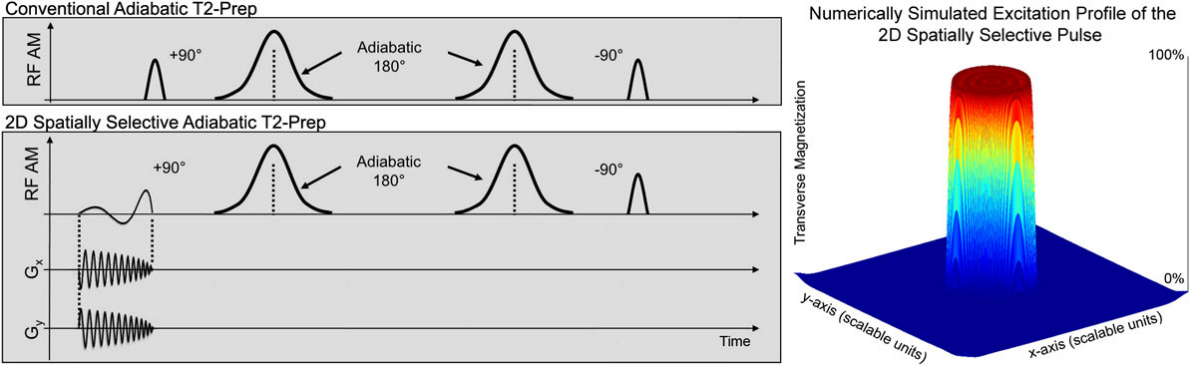
**Schematic representation of the 2D spatially selective adiabatic T2-Prep and the corresponding numerically simulated excitation profile**.

The first RF pulse of a +90°,180°,180°,-90° adiabatic T2-Prep was replaced with a jinc-shaped RF pulse and spiral gradients (Figure 1), which excites a cylindrical volume. Meanwhile, the final RF pulse (-90°) remains non-selective. It thus restores the excited cylinder, while simultaneously tipping down magnetization outside of the cylinder, which is then spoiled. After numerical simulations, phantoms were scanned (not shown), and volume targeted 3D images of the left coronary arterial system were acquired. In experimentally optimizing the protocol, 5 volunteers were scanned. All images were acquired on a 1.5T Siemens Aera using an ECG triggered and navigator gated 3D segmented k-space Cartesian gradient echo sequence, with FoV 432 × 288 mm, matrix size 288 × 192, 1.5 mm reconstructed slice thickness, 24 mm volume thickness, 8 k-space lines/heartbeat, 40 ms T2-Prep, 30° water selective RF excitation pulses, TE/TR/Tacq: 5.95/10.86/86.0 ms. Full FoV images were acquired with both the conventional T2-Prep and the 2D T2-Prep. The 2D T2-Prep scan was then repeated with signal foldover and a reduced FoV (rFoV: 120 × 120 mm, matrix size 80 × 80). All images were reformatted and analyzed using Soap-Bubble. The 3 T2-Preps (conventional, 2D, and 2D rFoV) were compared using vessel sharpness measurements, contrast-to-noise (CNR), and signal-to-noise (SNR) quantification in the blood and myocardium.

## Results

**Figure 2:**
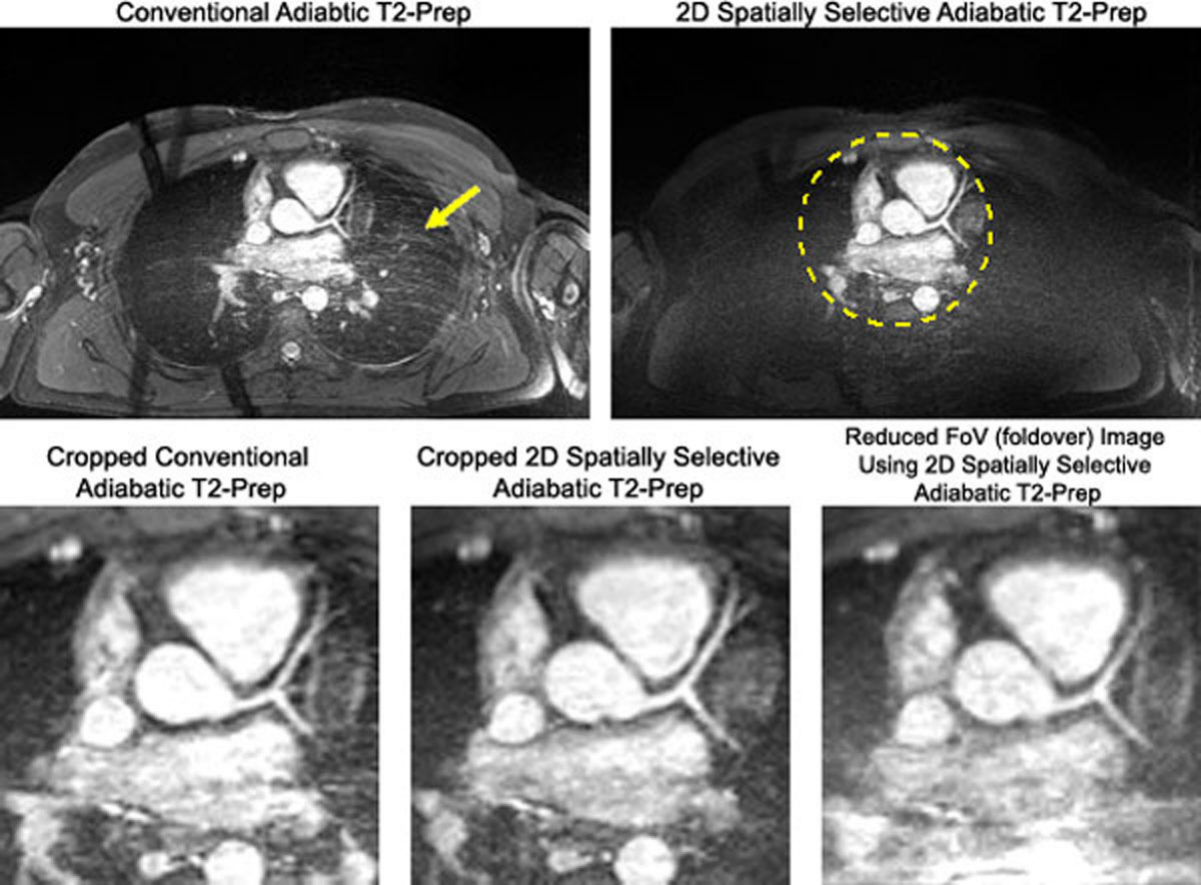
**Top Row: Comparison of conventional adiabatic T2-Prep and the 2D spatially selective adiabatic T2-Prep**. The region targeted by the 2D pulse is outlined by a dashed circle. Note the reduction in respiratory ghosting. Bottom Row: Comparison of cardiac anatomy using the conventional adiabatic T2-Prep, the 2D spatially selective adiabatic T2-Prep, and a reduced FoV image acquired using the 2D spatially selective T2-Prep.

The simulated excitation profile of the 2D pulse can be seen in Figure 1, with sample in vivo images shown in Figure 2. In comparing the T2-Preps, the 2D T2-Prep's mean blood and myocardium SNRs (580.2 and 191.2, respectively) stayed within the same range as the conventional T2-Prep's (606.1 and 191.4). The 2D T2-Prep also preserved blood-myocardium CNR (388.1) as compared to the conventional T2-Prep (415.8), demonstrating that the 2D T2-Prep adequately maintains T2-weighting. Vessel sharpness was also comparable (58.4% using the conventional T2-Prep vs. 58.5% using the 2D T2-Prep). In going to a reduced FoV, the mean blood and myocardium SNR decreased slightly (453.9 and 141.8), as did CNR (312.0) and vessel sharpness (56.4%), but scan time was reduced by 60%.

## Conclusions

A 2D T2-Prep shows promise to both reduce respiratory motion artefacts and decrease scan time, while preserving the T2-weighting of a conventional T2-Prep. It should thus be considered as a potential tool in accelerated cardiac imaging.

